# Cotinine inhibits TLR4/NF-κB signaling pathway and improves deep vein thrombosis in rats

**DOI:** 10.1042/BSR20201293

**Published:** 2020-06-04

**Authors:** Zhiyuan Cheng, Wei Jia, Xuan Tian, Peng Jiang, Yunxin Zhang, Jinyong Li, Chenyang Tian, Jianlong Liu

**Affiliations:** Department of Vascular Surgery, Beijing Jishuitan Hospital, Beijing City, China

**Keywords:** cotinine, TLR4/NF-κB signal pathway, venous thrombosis

## Abstract

**Background**: The present study was designed to explore the regulatory mechanisms and influences of cotinine on deep vein thrombosis (DVT) in rats via the toll-like receptor 4/nuclear factor κ binding (TLR-4/NF-κB) pathway. **Methods:** In this experimental study, 30 SD rats were randomly assigned to control group, sham operation group, model group, cotinine (10 μg/kg) group, and model + cotinine (10 μg/kg) group. The thromboxane B2 (TXB2), 6-keto-PGF_1α_, plasminogen activator inhibitor (PAI), tissue plasminogen activator (t-PA), TLR4, NF-κB, and p65 mRNA and protein expression and tissue changes were analyzed by ELISA, Hematoxylin–Eosin (HE) staining, RT-PCR, and Western blot. **Results:** There was no significant difference between the control and sham operation groups (*P*>0.05). The model and cotinine groups showed significantly higher mRNA and protein levels of TXB2, interleukin-6 (IL-6) and tumor necrosis factor-α (TNF-α), PAI, TLR*-4*, and NF-κB, and significantly lower levels of 6-keto-PGF_1α_ and t-PA than the control and sham operation groups (*P*<0.05), and the model + cotinine group showed significantly higher mRNA and protein levels of TXB2, IL-6 and TNF-α, PAI, TLR*-4*, and NF-κB and significantly lower levels of 6-keto-PGF_1α_ and t-PA than the model group (*P*<0.05). **Conclusion:** Cotinine can aggravate thrombus and inflammation in rats with DVT, and the mechanism may be associated with the activation of the TLR-4/NF-κB inflammatory signaling pathway.

## Introduction

Deep venous thrombosis (DVT) can give rise to serious cardiovascular disorders [1,2]. Generally, DVT can be caused by numerous risk factors including genetic factors, dietary habits, obesity, aging, trauma, and cancer [3,4]. Patients with acute DVT face a high risk of death [5]. Clinical investigation has previously shown that the incidence of DVT is related to sex, and the inferior vena cava thromboembolism is the main cause of proximal DVT [6]. The imbalance in coagulation and fibrinolysis system plays a crucial role in the pathogenesis of arterial thrombosis [7]. Despite extensive research, DVT remains a primary clinical disease. In the absence of preventive measures, the incidence of DVT was found to be 10–40% after internal or general surgery and 40–60% after major orthopedic surgery [8]. DVT can give rise to further disorders like pulmonary hypertension, repeated thrombosis, post-thrombotic syndrome, and even fatal pulmonary embolism. Surgical treatment may be an option, but it may destroy venous walls and vascular endothelial cells, and thereby increase the risk of thrombosis recurrence and activation of vascular smooth muscle cells, which often leads to poor prognosis and even further aggravates the disease [9,10].

Cotinine, with chemical formula C_10_H_12_N_2_O and a molecular weight of 176.22, can stimulate the nervous system [11]. It is the primary metabolite of nicotine *in vivo*, mainly present in blood and excreted via urine. The level of cotinine in serum can reflect the exposure level of smoking. Some studies have found that the level of cotinine was positively correlated with the occurrence of thromboangiitis obliterans, while others have found that high content of cotinine in serum is linked to an elevated risk of coronary heart disease [12–14].

Toll-like receptors (TLRs) and its family member TLR4 are a kind of natural immune receptor [15]. TLR4 signaling pathway is involved in the process of thrombosis, and TLR4 is able to identify pathogen-associated molecular patterns and damage-associated molecular patterns and is a cell surface sensor. The interaction of TLR4 receptors triggers the activation of downstream pathways, resulting in phosphorylation and translocation of downstream factors [16]. Activated TLR4 induces the late activation of nuclear factor κ binding (NF-κB) and the induction of inflammasomes as well as promotes the activation of leukocytes and the intrinsic renal cells in non-immune nephropathy [17,18]. NF-κB controls many biological processes, such as inflammation and apoptosis [19]. For example, in tumor necrosis factor-α (TNF-α)-induced cell apoptosis, NF-κB can significantly increase TNF-α expression and cause widespread inflammation [20]. TLR4, thus, mediates the activation of NF-κB signaling pathway and strongly affects the uncontrolled inflammatory response [21]. It is essential to understand the relationship between NF-κB and various downstream signaling molecules. Many signaling molecules can activate NF-κB, but when without regulated function, NF-κB can become the driving force of a disorder [22]. Some studies have found that as the serum cotinine levels increase, the incidence of thromboangiitis obliterans is also increased. In addition, TLR4 expression in human umbilical vein endothelial cells treated with cotinine increased significantly, indicating that cotinine activates TLR4 receptor [23]. However, there are no relevant reports on whether cotinine participates in the development of DVT by mediating the TLR4/NF-κB signaling pathway.

Therefore, in the present study, a rat model of DVT was established, and the effects of cotinine on DVT in rats were elucidated through *in vivo* experiments and various molecular biological techniques.

## Materials and methods

### Reagents and consumables

SDS/PAGE reagents, Protease Inhibitor, BCA Protein Concentration Kit (Biosharp Company); Electrophoresis Instrument (EPS 300, Bole Bio-Rad Company, U.S.A.); Microplate Reader (Multiskan MK3, Thermo Instrument Equipment Co., Ltd., U.S.A.); Tissue Homogenator (Haimen Aiband Laboratory Equipment Co., Ltd., China); qPCR Instrument (7900 Fast, Applied Biosystems, U.S.A.); TRIzol reagent, DEPC water, Medical Discovery Leader, UltraPure Agarose, SuperScript III RT reverse transcription kit, SYBr qPCR mix (ABI), pipettor (Eppendorf); ELISA test kits of interleukin-6 (IL-6), TNF-α, thromboxane B2 (TXB2), and 6-keto-PGF_1α_ (Nanjing SenBeiJia Biological Technology Co., Ltd., China); rabbit anti-TLR-4, phospho-NF-κBp65 (Ser^468^), rabbit anti-NF-κBp65 antibody and horseradish peroxidase (HRP)-labeled goat anti-rabbit secondary antibody (Bioss Biotechnology Co., Ltd., Beijing, China). All operations involving animals were carried out under the ethical standards of Beijing Jishuitan Hospital (No. 2018060009) at which the studies were conducted. All of the above experiments were carried out with permission from the Animal care and Use Committee of Beijing Jishuitan Hospital.

### Establishment and grouping of animal models

All experiments involving animals in the article were carried out at the Beijing Jishuitan Hospital Animal Laboratory. In this experimental study, deprived of food and drinking water for 12 h, male SD rats were injected with 1% pentobarbital sodium for anesthesia before surgery. They were immobilized in a supine position on the operating table. The skin was incised in the midline of the abdomen. To establish a rat model of DVT, the inferior vena cava was separated and the left femoral vein near the heart was ligated under the left renal vein with thick silk thread. Operations for the normal group and the cotinine group were the same with those for other groups except ligation. The cotinine and model + cotinine groups were given 10 μg/kg cotinine solution (Sigma, U.S.A.) per day by gavage while the other groups were given saline of the same volume. Each group consisting of six rats were continuously fed for 2 weeks. At the end of the experimental period, blood and venous tissue were sampled from each group of rats. Some of the tissues were stored in 4% paraformaldehyde for Hematoxylin–Eosin (HE) staining while some were stored in −80°C refrigerator for determination of the gene and protein expression. At the end of the animal experiment, the cervical spine dislocation method was used to kill the experimental animals. The specific steps were as follows: grasp the mouse tail with your right hand and pull it backwards, while holding down the mouse head with the thumb and index finger of your left hand to pull the spinal cord and brain spinal cord.

### Detection of serum TXB2 and 6-keto-PGF_1α_, inflammatory factors, plasminogen activator inhibitor and tissue plasminogen activator by ELISA

Serum TXB2 and 6-keto-PGF_1α_ levels, inflammatory factors, plasminogen activator inhibitor (PAI) and tissue plasminogen activator (t-PA) were detected by ELISA. Rat-tail vein blood (4 ml) was collected aseptically and centrifuged at a low temperature at 3000×***g*** for 10 min. The supernatant was collected and separated into 200 μl centrifuge tubes. The tested samples (100 μl) were incubated at 37°C and cleaned after 60 min. The changes in each index were measured by the kits, and the experiment was carried out in accordance with the kit instructions. The absorbance of TXB2 and 6-keto-PGF_1α_, inflammatory factors, PAI and t-PA in each group was measured by microplate reader.

### HE staining to observe the changes in venous tissue

After pentobarbital anesthesia, rats from each group were killed aseptically. The vein tissues were isolated and the dissected vein tissues were immersed in formalin. Then the vein tissues were washed with running water for 24 h and then embedded with paraffin after being hyalinized and dipped in wax. The embedded block was cut into pathological sections approximately 5-μm-thick, stained with Hematoxylin for 15 min, washed with water, re-stained with Eosin solution for 5 min, dehydrated with alcohol, hyalinized, sealed with neutral resins, and finally evaluated under a light microscope.

### Detection of related gene expression by RT-PCR

(1) After pentobarbital anesthesia, rats from each group were killed aseptically, and vein tissues of the right lower extremity were separated. These tissues were weighed 100 mg carefully and accurately at a low temperature, ground using liquid nitrogen, and then homogenized with lysis buffer at a low temperature at 2200 rpm for 15 s. Total RNA was extracted from the tissues, and the RNA concentration was estimated. (2) The amplification was carried out in a 20-μl system (cDNA (2 μl), master-mix (10 μl), primer (2 μl), ddH_2_O (6 μl)) for 40 cycles, generating the mRNA to cDNA. (3) After cDNA synthesis, PCR amplification was carried out as follows: Pre 95°C for 2 min, 94°C for 20 s, 60°C for 20 s, and 72°C for 30 s, for 40 cycles. The target gene and GAPDH internal reference sequence were designed according to the GenBank sequence, and the expression of the target gene was detected by qRT-PCR. Relative expression levels of related genes in vein tissues of right lower extremity of rats in each group were calculated by the 2^−ΔΔ*C*_t_^ method [24].

### Western blot assay

(1) After pentobarbital anesthesia, rats in each group were killed aseptically, and vein tissue of right lower limb was separated. The right lower limb vein tissue (150 mg) was accurately weighed and put in a 10-ml EP tube. After grinding at a low temperature, the tissue was quickly homogenized by a homogenizer and centrifuged to collect the supernatant. (2) The supernatant was collected and transferred to the EP tube, i.e. the total protein solution. Loading dye was added to the total protein solution and the mixture was boiled for 10 min. The protein concentration was detected in accordance with the instructions on BCA kit. (3) According to the protein concentration, 30 μg loading protein was loaded on to each well for SDS/PAGE. (4) The proteins on the gel were transferred to a PVDF membrane at 300 mA (constant current), and the membrane transfer was stopped after 1.5 h. (5) According to the marker, the target band was collected and incubated with the blocking solution. Then, TLR-4 (1:1000), Phospho-NF-κBp65 (Ser^468^) (1:1000), rabbit anti-NF-κBp65 antibody (1:1000), GAPDH (1:1000) primary antibodies were added and incubated. (6) In the next morning, the target band was washed with TBST solution three times, 5 min each time. HRP-labeled secondary antibody solution was put and cultured at indoor temperature for 1 h. (7) DAB solution was used for color rendering, the level of protein to be measured was corrected, and Image Lab Software was used to detect the gray-scale value of the protein band. The protein expression = the gray-scale value of the target protein/the gray-scale value of GAPDH.

### Statistical analyses

The data were processed by SPSS 21.0 software. One-way ANOVA (F test) was employed for multigroup comparison; if *P*<0.05, LSD t test was applied for intergroup comparison. The experimental results were expressed as the mean ± standard deviation (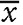 ± sd). *P*<0.05 indicates a significant difference. The histogram was drawn using GraphPad Prism 8.0.

## Results

### Serum TXB2 and 6-keto-PGF_1α_ levels

There was no significant difference between the sham-operation group and the control group. The model group and the cotinine group showed higher serum TXB2 level and lower serum 6-keto-PGF_1α_ level than the control group and the sham operation group (all *P*<0.05), and the model + cotinine group also showed higher TXB2 level and lower 6-keto-PGF_1α_ level than the model group (all *P*<0.05).

### Inflammatory factors in rats of each group

There was no significant difference in inflammatory factors between the sham operation group and the control group. The IL-6 and TNF-α expression in the veins of the right lower extremity of rats in the model and cotinine groups was significantly higher than that in the control and sham operation groups (all *P*<0.05), and the expression of IL-6 and TNF-α in the right lower limb vein of rats in the model + cotinine group was also significantly higher than the model group (both *P*<0.05).

### Fibrinolytic indices PAI and t-PA

There was no significant difference in PAI and t-PA levels between the control group and the sham operation group. The model group and the cotinine group showed significantly lower t-PA level and significantly higher PAI level than the control group and the sham operation group (all *P*<0.05). In comparison with the model group, t-PA in the serum of rats in model + cotinine group was significantly lower and PAI was significantly higher (both *P*<0.05).

### HE staining to observe the changes in venous tissue

**Figure 1:**
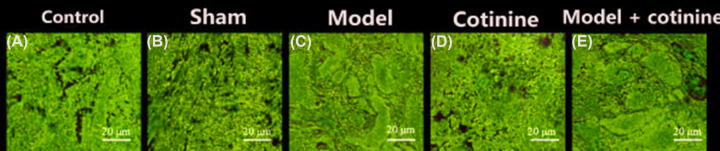
HE dyeing experiment pictures (**A–E**) HE staining (200×). (A) Control group, (B) Sham operation group, (C) Model group, (D) Cotinine group, (E) Model + Cotinine group.

The control group (Figure 1A) and the sham operation group (Figure 1B) were shown in Figure 1. The Model group (Figure 1C) showed that the wall of the inferior vena cava was shed due to complete necrosis. The wall of the inferior vena cava in the cotinine group had partial necrosis (Figure 1D). In the model + cotinine group (Figure 1E), the vascular cavity was significantly dilated and the contour of the tube wall was unclear, which made it difficult to identify.

### Detection of related gene expression by RT-PCR

**Figure 2:**
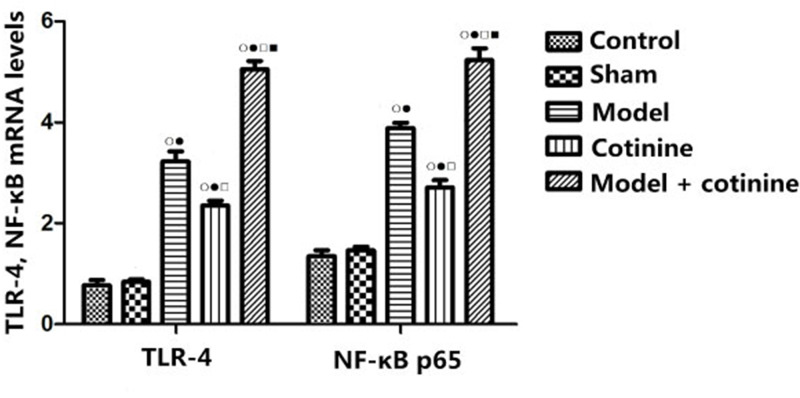
The expression levels of related genes detected by RT-PCR Compared with control group, ^ο^*P*<0.05; compared with sham operation group, ^•^*P*<0.05; compared with model group, ^□^*P*<0.05; compared with cotinine group, ^▪^*P*<0.05.

As shown in Figure 2, there was no significant difference in gene expression between the sham operation and the control groups. The mRNA levels of TLR-4 and NF-κB in the lower limb vein tissues in the model group and the cotinine group were significantly higher than those in the control group and the sham operation group (all *P*<0.05), and the mRNA levels of TLR-4 and NF-κB in the model + cotinine group were also significantly higher than those in the model group (both *P*<0.05).

### Western blot detection of the expression of related proteins

**Figure 3:**
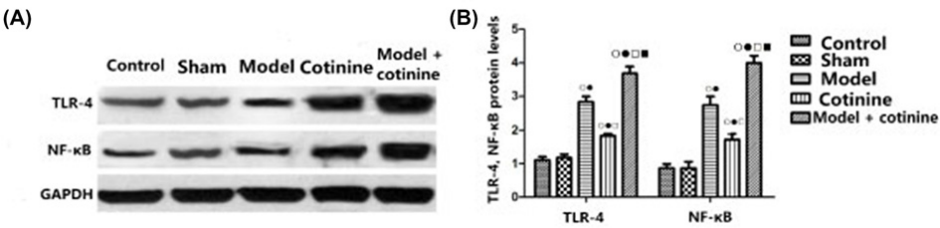
Western blot detection. (**A**) Protein bands, (**B**) protein expression levels. (B) Compared with control group, ^ο^*P*<0.05; compared with sham operation group, ^•^*P*<0.05; compared with model group, ^□^*P*<0.05; compared with cotinine group, ^▪^*P*<0.05.

As shown in Figure 3, there was no significant difference in protein expression between the sham operation group and the control group. The levels of TLR-4 and NF-κB (Ser^468^) proteins in the lower limb veins of rats in the model group and the cotinine group were significantly higher than those in the control group and the sham operation group (all *P*<0.05), and the protein levels of TLR-4 and NF-κB (Ser^468^) in the lower limb venous tissues of rats in the model + cotinine group were also significantly higher than those in the model group (both *P*<0.05).

## Discussion

DVT impacts approximately 250000 patients each year, showing a high morbidity and mortality. At present, anticoagulants including warfarin, heparin, aspirin, and clopidogrel can strongly inhibit the progression and recurrence of DVT, but they bring about a high risk of internal bleeding [25]. Vein wall injury, blood stasis, and hypercoagulability are risk factors for DVT. They are closely related to the inflammatory process, involving the aggregation and adhesion of white blood cells such as neutrophils. Thrombosis induces the release of cytokines including IL-6, tissue factor, and TNF-α by activating endothelial cells, and inflammation accelerates hypercoagulability state and leads to endothelial damage and platelet aggregation, thus further aggravating the development of DVT [26]. The imbalance in the coagulation and fibrinolysis system is important in the development and pathogenesis of arterial thrombosis. In the present study, we established a rat model of DVT and observed that serum TXB2 level increased in the model group while the 6-keto-PGF_1α_ level declined. The same results were observed in the cotinine group, but the relative change was smaller compared with the model group. The serum TXB2 level elevated more significantly in the model + cotinine group.

The IL-6 and TNF-α levels in the veins of the right lower extremity of rats in the model group elevated significantly. The IL-6 and TNF-α levels in the right lower limb vein of rats in the model + cotinine group was significantly higher than those in the model group. In addition, lower limb tissue injury was serious in the model group and was more advanced in the model + cotinine group. And the inferior vena cava wall from the model group disappeared due to complete necrosis. In the model + cotinine group, the vascular lumen was dilated and the wall was unclear and difficult to recognize. These results implied that there was severe inflammation and histopathological damage in rats with DVT. After treatment with cotinine, the expression level of inflammatory factors increased significantly, above that of non-cotinine group. Studies have found that the concentration of cotinine was positively correlated with inflammatory factors and pathological changes in experimental rats. A higher concentration of cotinine resulted in higher expression of inflammatory factors, and more serious tissue damage. Our study results are similar to those observed in the previous studies [27–29].

The interaction of TLR4 receptors triggers the activation of downstream pathways, which leads to phosphorylation and translocation of downstream factors and in turn causes late activation of NF-κB and induction of inflammatory bodies, and finally makes the body subject to the toxic reaction of TNF-α [30]. In addition, the NF-κB transcription factor regulates the expression of tissue factor in DVT [31]. The results of gene expression showed that the mRNA levels of TLR-4 and NF-κB in serum elevated in the model group as well as in the cotinine group, whose fold change was smaller than that of the model group. The mRNA levels of TLR-4 and NF-κB in model + cotinine group increased more significantly as compared with other groups. Our results indicated that the effect of cotinine on DVT in rats was due to activation of the TLR-4/NF-κB signaling pathway, which revealed the specific role of cotinine in DVT in rats. Our results are similar to those seen in previous studies, which provides evidence that cotinine aggravates inflammation through TLR-4/NF-κB [32,33].

In the present study, there are some limitations. Different doses of cotinine have not been tested, but different concentrations of cotinine may have varied effects on venous thrombosis in rats. We have also not studied the effect of cotinine on rats after TLR4 or NF-κB inhibitors were used. Lack of time, the rats have not been tested for other diseases before the experiment, which may affect the detection indicators. Besides, this group of experiments have utilized only one drug treatment. More experimental methods (immunohistochemistry, immunofluorescence) should be added in the future studies for validating the results in the present study.

A range of pathological changes including inflammation and abnormal hemorheological factor expression may occur in DVT. Cotinine can further aggravate DVT through the TLR-4/NF-κB signaling pathway and can be a new target for the therapy of DVT. In short, the present study provides a basis for the prevention and treatment of DVT and provides new avenues for further research.

## Conclusion

In the present study, there were some limitations. Different doses of cotinine were not tested, but different concentrations of cotinine could have varied effects on venous thrombosis in rats. We also did not study the effect of cotinine on rats after TLR4 or NF-κB inhibitors were used. Lack of time, the rats were not tested for other diseases before the experiment, which may affect the detection indicators. Besides, this group of experiments utilized only one drug treatment. More experimental methods (immunohistochemistry, Immunofluorescence) should be added in the future studies for validating the results in the present study.

## Abbreviations

EP: eppendorf
GAPDH: glyceraldehyde-3-phosphate dehydrogenase
HE: Hematoxylin–Eosin
HRP: horseradish peroxidase
IL-6: interleukin-6
LSD: least significant difference
NF-κB: nuclear factor κ binding
PAI: plasminogen activator inhibitor
SD: Sprague-Dawley
TLR: toll-like receptor
TNF-α: tumor necrosis factor-α
TXB2: thromboxane B2
t-PA: tissue plasminogen activator
qRT-PCR: quantificational rt-PCR
